# Encapsulated Carbenoxolone Reduces Lung Metastases

**DOI:** 10.3390/cancers11091383

**Published:** 2019-09-17

**Authors:** Adi Karsch-Bluman, Shimrit Avraham, Miri Assayag, Ouri Schwob, Ofra Benny

**Affiliations:** 1 The Institute for Drug Research, The School of Pharmacy, Faculty of Medicine, The Hebrew University of Jerusalem, 9112102 Jerusalem, Israel; adikarsch@gmail.com (A.K.-B.); ouris@ekmd.huji.ac.il (O.S.); 2 Department of Cell Biology and Cancer Science, The Ruth and Bruce Rappaport Faculty of Medicine, Technion—Israel Institute of Technology, Efron 1, 3535422 Haifa, Israel; shimritiavra@gmail.com; 3 The Institute of Pulmonary Medicine at Hadassah-Hebrew University Medical Center, 9112102 Jerusalem, Israel; miriag1@gmail.com

**Keywords:** cancer, carbenoxolone, lungs, metastases, particles, drug delivery, double emulsion, intranasal administration

## Abstract

Carbenoxolone is an anti-inflammatory compound and a derivate of a natural substance from the licorice plant. We previously showed that carbenoxolone reduces the metastatic burden in the lungs of mice through its antagonistic effect on high mobility group box 1 (HMGB1). To further enhance carbenoxolone’s activity and localization in the lungs, thereby reducing the potential adverse side effects resulting from systemic exposure, we developed a poly(lactic-co-glycolic acid) (PLGA) slow-release system for pulmonary delivery which maintains drug activity in-vitro, as demonstrated in the anoikis assay. Both systemic and intranasal administrations of carbenoxolone effectively minimize metastatic formation in a lung colonization model in mice. Our results show a decrease in the metastatic burden in the lung tissue. Notably, the therapeutic effect of a single intranasal administration of 25 mg/kg carbenoxolone, in the form of drug-loaded particles, had a similar effect in reducing metastatic lesions in the lungs to that of a 10-fold dose of the free drug via intraperitoneal injections, three times per week over the course of four weeks. These data offer new means to potentiate the anti-cancer activity of carbenoxolone and simultaneously reduce the requirement for high dosage administration; the upshot substantially improves therapeutic effect and avoidance of side effects.

## 1. Introduction

Lung cancer is one of the deadliest diseases. This is primarily due to the late diagnosis, which results from a lack of symptoms in the early stages, and which evidently promotes high mortality rates [1,2,3]. Even among patients diagnosed at an early stage, metastases frequently develop, and therapeutic options are, unfortunately, rather limited [4,5]. Therefore, in addition to the ongoing efforts for early lung cancer diagnosis, metastasis prevention is of great importance.

Despite the increasing number of personalized targeted therapies for specific genetic profiles, and the availability of over 60 FDA-approved drugs for treating lung cancer [6], prophylactic therapy for lung metastases is not yet available. Patients diagnosed with early-stage lung cancer, or those with primary tumors that are known to be susceptible to developing lung metastases could potentially benefit from such therapy, which could prevent further progression of the disease. 

In our recently published work, we demonstrated that carbenoxolone, a derivate of a natural compound, can significantly diminish the metastatic burden in the lungs of mice [7,8]. Carbenoxolone is a water-soluble-small-molecule that is chemically derived from glycyrrhizin, an anti-inflammatory active compound found in the licorice root extract. Carbenoxolone is a licensed drug in the UK, prescribed for inflammation and esophageal ulcers [9]. It was found to be an antagonist of endotoxin-induced secretion of high mobility group box 1 (HMGB1) [10], a known pro-inflammatory protein. One of the receptors of HMGB1 is the receptor for advanced glycation end-products (RAGE), which is highly abundant in the lung tissue. As demonstrated here, applying both in-vitro and in-vivo models, carbenoxolone has the capacity to antagonize HMGB1 and to inhibit lung cancer colonization. Interference of carbenoxolone in cancer colonization was found to be mediated by impairment of cell adhesion via reduction of intercellular adhesion molecule 1 (ICAM1) expression levels, thus diminishing cell-cell interactions and cellular adherence to the extracellular matrix (ECM) [8].

Although prescribed for gastrointestinal inflammation, carbenoxolone carries potential adverse side effects under systemic exposure to high dosage. The common dosage is 100 mg for one week, three times a week, followed by a reduction to 50 mg every other day, to prevent potential side effects [11] such as sodium retention, hypokalemia [12], increased blood pressure, and edema [13]. Localizing the effect of the drug to the organ-of-interest would decrease the required dosage, thus provide more effective treatment, with fewer side effects and increased patient compliance. 

Here we show our development of a pulmonary slow-release system containing carbenoxolone. Carbenoxolone-loaded biodegradable poly(lactic-co-glycolic acid) (PLGA) particles were fabricated, optimized, and characterized. The drug carrier was designed to localize drug activity in the lung, and enable the steady and continuous release of the drug. Repurposing and reformulating carbenoxolone to prevent lung metastases, offer a promising direction for clinical use. In a recently published study [7], we showed that combined treatment of carbenoxolone with Bevacizumab (Avastin), an anti-vascular endothelial growth factor (VEGF) drug, prolonged the efficacy of the drug and resulted in a significantly lower tumor volume. The method of drug delivery, as presented in the current study may be extended to include different drug combinations and to serve as a platform for simultaneously treating and preventing cancer progression. 

## 2. Results

### 2.1. Carbenoxolone Depicts Low Stability in an Aqueous Solution

**Figure 1:**
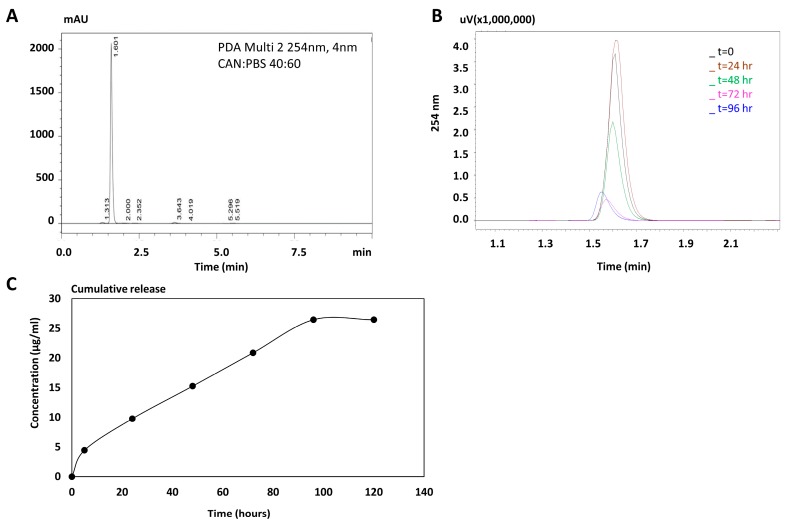
Stability of carbenoxolone and release from poly(lactic-co-glycolic acid) (PLGA) particles using HPLC. (**A**) HPLC detection of carbenoxolone in its free form; (**B**) evaluation of carbenoxolone in an aqueous solution over 96 h; and (**C**) a cumulative release of carbenoxolone from PLGA particles over 96 h.

Prior to encapsulation we aimed to monitor the stability of free carbenoxolone in an aqueous solution, as this compound is of high water- solubility [14]. High-performance liquid chromatography (HPLC) results of samples that were taken from the original stock of 2 mg/mL, showed a rapid decrease of 40% in the intensity of the peak (254 nm), after merely 48 h (Figure 1B). The peak obtained 72 h after introduction of the drug to the solution, was 85% lower than that found at t = 0. This demonstrates relatively low stability of the free drug in an aqueous solution. 

### 2.2. Tween 80 Affects Particle Size and Morphology

**Figure 2:**
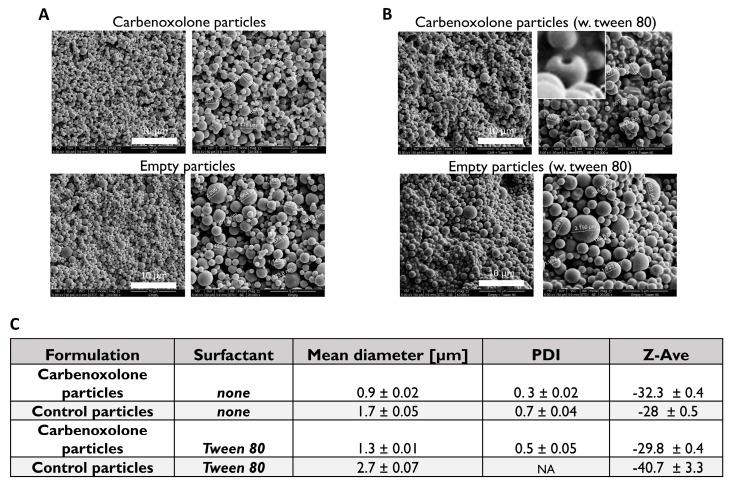
Fabrication of carbenoxolone-loaded particles. Effect of surfactant (Tween 80) on the size of particles. (**A**) Scanning electron microscope (SEM) images of PLGA particles fabricated by double emulsion. Without surfactant, particles are of smooth and spherical shape; (**B**) the effect of the addition of surfactant on the mean diameter and size distribution of particles; and (**C**) a summary of the effect of surfactant on drug encapsulation and the size of particles.

Morphology of the PLGA particles which were formulated by the double-emulsion technique was analyzed using a scanning electron microscope (SEM), and their size and charge were determined by Dynamic Light Scattering (DLS)/Zeta sizer. Aiming at a small-sized, uniform, spherical shaped particle, two fabrications were compared: one with Tween 80 and one without an added surfactant (Figure 2, Figure S1). Figure 2B demonstrates the result of the addition of Tween 80 to the formulation that was added to stabilize the primary oil-in-water (O/W) emulsion. The presence of Tween 80 promoted the formation of slightly larger particles compared with those formulated without the addition of the surfactant. However, when Tween 80 was added to the formulation with the drug, the formed particles had a spherical shape with a ‘hollow-like’ core. All the drug-free formulations yielded particles of a plain spherical shape with smooth surface. Figure 2 shows the effect of both carbenoxolone and Tween 80 on particle size and morphology. Interestingly, the particles with the drug were smaller than the empty particles. Based on both the shape and size of the particles, we decided to continue our investigation using the particles without Tween 80. 

### 2.3. Carbenoxolone Is Successfully Encapsulated in Polymeric Particles

Carbenoxolone loading in PLGA particles was determined using HPLC (Figure 1C). Drug encapsulation was estimated at 60% (*w/w*). Our calculations were based on loading of 2.65 µg carbenoxolone per 1 mg of dry particles as assessed after 96 h, and multiplying by the percentage of disintegration (Figure 1C) as determined in the stability test (For calculations see Appendix A). 

### 2.4. The Activity of Carbenoxolone Loaded Particles Is Maintained in Anoikis Assay

**Figure 3:**
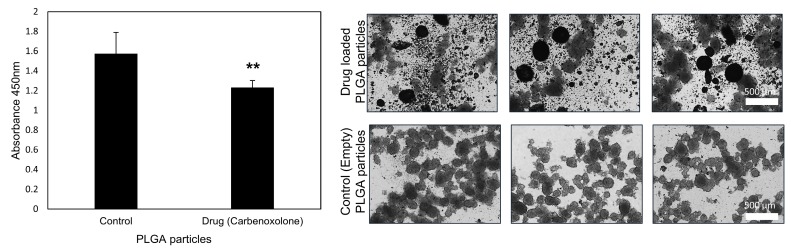
Carbenoxolone-loaded particles increase cell susceptibility to anoikis. Anoikis assay using poly(2-hydroxyethyl methacrylate) (p-HEMA) coated plates on Lewis lung carcinoma (LLC), after 72 h of incubation with 0.5 μM carbenoxolone encapsulated particles (empty particles for control). n = 5.

We previously found that carbenoxolone is highly active in the anoikis assay [8]. Since cell survival in blood circulation is a key factor in the metastatic cascade, we established that the main effect of the drug is on cell-cell interactions, and showed that exposure to carbenoxolone increases the susceptibility of cancer cells to anoikis [8]. In our previous study [8] the anti-cancer activity of carbenoxolone was examined using the Lewis lung carcinoma (LLC) cell line, due to the ability of their primary lung tumors to metastasize to secondary sites in the pulmonary tissue or other distant organs. This study was performed based on our earlier work; hence we continued our investigation using the same cell line. To evaluate the effect of carbenoxolone loaded particles, and to confirm the activity of the formulation in its particulate form, we examined the level of resistance to apoptosis of non-adherent LLC cells treated with drug-loaded particles for 72 h, by detecting cell viability on a non-adherent surface using WST8 (Figure 3). We found that cells treated with empty particles had higher survival rates than those treated with drug-loaded particles. Figure 3 indicates increased susceptibility to cell death as a result of drug-loaded particle treatment, as presented by a lower level of cell viability by approximately 22%. 

### 2.5. Carbenoxolone Loaded Particles Reduce the Number and Size of Metastases in the Lungs via the Tail Vein Model

**Figure 4:**
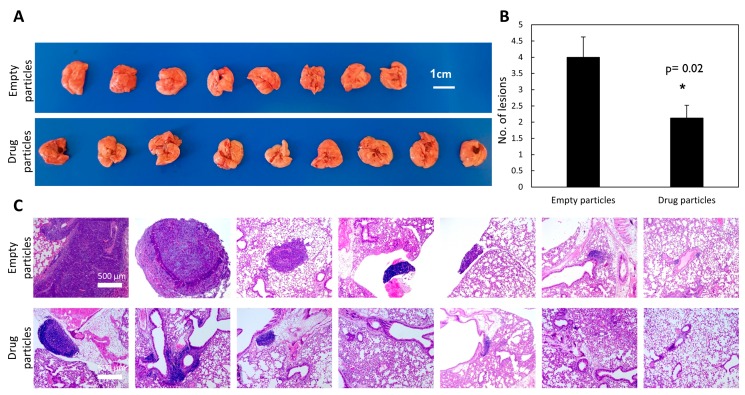
The effect of carbenoxolone-loaded particles in tail vein injection (40 mg/kg, single administration). (**A**) Tail vein systemic injections in C57BL/6 mice. Mice were pre-treated intravenously (IV) with drug-loaded particles (empty particles as control). On the 5th day, all mice were injected IV with Green Fluorescent Protein (GFP) tagged LLC cells (5 × 10^6^). The endpoint was 21 days after treatment initiation, and the lungs were resected. (**B**) The number of lesions found in the lungs of control mice (empty particles) compared with lesions found in the lungs of mice treated with carbenoxolone loaded particles. *p* = 0.02. (**C**) Representative histologic sections of lungs of control (empty particles- top) and carbenoxolone treated mice (drug-loaded particles- bottom) after the tail vein experiment post hematoxylin and eosin (H&E) staining (10×). n = 8–10. * *p* ≤ 0.05.

To investigate the effect of the drug-loaded particles, and to examine if they can diminish both the number and size of lesions in the lungs, as we previously demonstrated in the free drug [8], we studied colonization of cells in the lungs. For this purpose, we performed tail-vein injections of drug-loaded particles, followed by LLC injections in C57BL/6J mice 5 days later. Three weeks after cell injections, the experiment was terminated, mouse lungs were removed, and histological sections were stained with hematoxylin and eosin (H&E) to assess the burden of the lesions. Tissue analysis showed that carbenoxolone reduced both the number and size of the lesions (Figure 4). We used a scoring system based on lesion size as follows: large (>500 µm), medium (250–500 µm), and small (<250 µm), lesions in each tissue section. The mean numbers of lesions found in the lungs of the control and treated groups, respectively, were 32 vs. 17, with a size distribution of: large 7 and 3; medium 17 and 9; small 8 and 5 (average of nine slides per group). The results show significantly lower metastatic formation in the lungs of mice that were pretreated intravenously (IV) with carbenoxolone loaded particles, than in the lungs of those treated with the empty particles (*p* = 0.02). 

### 2.6. Intranasal Administration of Carbenoxolone Particles Significantly Lowers Metastatic Burden in the Lungs of Mice

**Figure 5:**
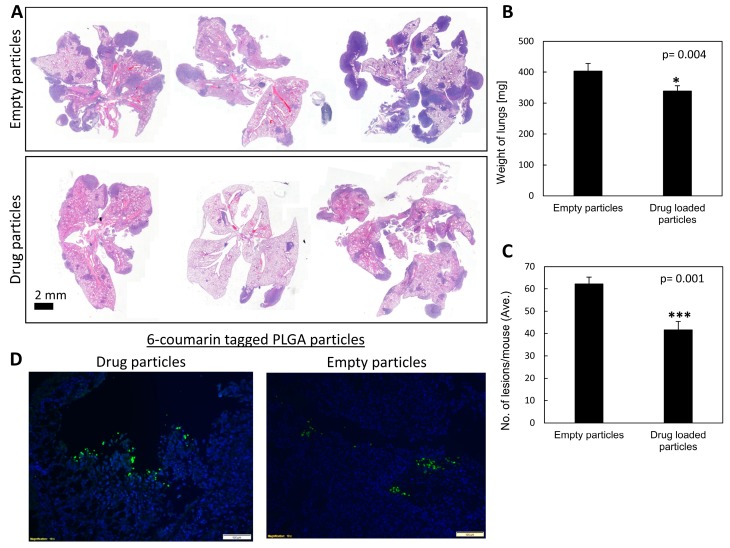
The effect of carbenoxolone loaded particles resulting from intranasal administration (1 mg/mouse, single administration). C57BL/6J mice were pre-treated intranasally with 6-coumarin drug-loaded particles, or with empty particles as control. On the 5th day, all mice were injected intravenously with Lewis lung carcinoma (LLC) cells (5 × 10^6^). The endpoint was 20 days after treatment initiation due to death of mice in the control groups, after which lungs of all mice were resected. (**A**) Representative hematoxylin and eosin-stained histological sections of lungs removed from untreated (empty particles- top) or treated groups (10×). (**B**) The weight of lungs of untreated mice compared with that of mice treated with carbenoxolone loaded particles, *p* = 0.004. (**C**) The number of lesions found in the lungs of untreated mice compared with the number found in the lungs of mice treated with carbenoxolone loaded particles, *p* = 0.0057. (**D**) Representative pictures showing that 6-coumarin tagged particles target the lungs when administered intranasally, with and without drug encapsulation. *n* = 9–10. * *p* ≤ 0.05, *** *p* ≤ 0.001.

To better target treatments to the pulmonary tissue, particles were introduced to mice in a single intranasal administration. C57BL/6J mice were injected IV with LLC cells 5 days following treatment initiation and sacrificed 20 days later. Sectioning and staining the lungs for H&E showed that mice treated with the drug-loaded particles had developed 33% less metastatic lesions than the control group (Figure 5A,C). The mean numbers of lung lesions in the control and treated groups, respectively, were 62 vs. 42, with a size distribution of: large 24 and 14; medium 15 and 12; small 23 and 16. The PBS control showed results similar to those of the empty particle control (Table S1). These results show significantly lower metastatic formation in the lungs of mice intranasally treated with carbenoxolone loaded particles compared with lungs of mice that were treated with empty particles (*p* = 0.003). Additionally, the weight of lungs in mice that were treated with the drug encapsulated particles was significantly lower (*p* = 0.004) than that of the control group, and correlated with the lower number of LLC lesions (Figure 5B,C and Table S1).

### 2.7. Fluorescent Labeled Particles Are Detectable in the Lung Post Intranasal Administration

Fluorescent labeled particles are used to follow tissue distribution in-vivo. Figure 5D shows that both drug-loaded and empty particles reached the lungs and remained in the tissue as long as 3 weeks post single intranasal administration. The 6-coumarin tagged PLGA particles, either with or without the drug, accumulated in the lung tissue as visualized using fluorescent microscopy in histological sections (Figure 5). 

## 3. Discussion

Metastases are responsible for over 90% of cancer-related deaths, therefore is an important target for cancer therapy [15]. There is currently no available standard preventative treatment, and first-line therapy is commonly non-selective broad cytotoxic chemotherapy. Optimal prophylactic therapy would require a high safety profile and targeted non-invasive administration, to provide low systematic exposure over time that would maintain drug efficacy with minimal toxicity. 

In a recently published study, we showed that carbenoxolone possesses anti-cancer properties mediated by its antagonistic effect on HMGB1, as demonstrated in mice that were pre-treated with the drug [8]. This activity is highly relevant to the lungs, as this tissue expresses exceptionally high basal levels of RAGE, the receptor for HMGB1 [10]. The rationale of using pre-treatment is based on our former observation of carbenoxolone to reduce tumor colonization in the lung via the reduction of ICAM1 [8]. This finding suggests a possible clinical scenario in which carbenoxolone treatment can be prescribed as a prophylactic treatment to reduce the metastatic burden immediately after a diagnosis of a primary tumor with susceptibility to spreading into the lungs.

Carbenoxolone is known to provoke fluid retention, one of its dose-related side-effects and the main reason for its limited prescription [16,17]. To achieve the maximal therapeutic effect, together with minimal side-effects, carbenoxolone is usually given at a dose of 100 mg three times daily for one week, followed by 50 mg three times daily for up to 12 weeks. The lower dose provokes fewer side-effects but also provides a lesser effect [11].

In addition to sodium retention and hypokalemia [12], increased levels of carbenoxolone in the serum have been shown to be correlated with a drop in the levels of potassium in the blood, which could increase blood pressure and promote edema [13]. As expected, patient age was found to be associated with side-effects. This was attributed to the decreased rates of plasma clearance in elderly patients [18]. Therefore, elderly patients and patients with renal or liver diseases are prescribed lower than usual dosages [11]. 

The equivalent dose of 40 mg/kg was selected in our study based on previous experiments which identified 60 mg/kg dose in mice as the maximum tolerated dose, leading to a >10% weight loss in nude mice [8]. A dose of 50 mg/kg for 8 days with the free drug was less toxic, showing 6% of weight loss compared with 3% weight gain in the control mice (Table S3). Further in-depth toxicity studies should be performed in the future.

To provide locally targeted treatment, rather than a systemic treatment, we propose introducing carbenoxolone in a pulmonary targeting formulation, as this easier application could increase patient compliance [19]. In mice, biodegradable poly(sebacic anhydride) (poly(SA)) particles [20] and siRNA particles (siNS1) [21] were shown to penetrate the lung after intranasal delivery. In humans, the design can be further optimized to target the lungs, since both particle size and density affect the deposition of particles in this tissue [22,23]. Previous publications demonstrated that to reach the smaller airways in the lung, small (micron range) particles should be used [24]. The mechanism of these small particles is diffusion, as opposed to gravitation which is the mechanism of large particles [19]. 

A common means of drug encapsulation in PLGA spheres is the solvent evaporation method, by which water-soluble or insoluble drugs can be encapsulated using water-in-oil-in-water (W/O/W) double emulsion [25,26]. PLGA is a biodegradable biocompatible copolymer of lactic and glycolic acids that can be fabricated into various shapes and sizes [25,27,28] and is used in several FDA approved products [27]. PLGA has been widely studied in controlled-release systems for small molecules, and also with peptides and hormones [29,30,31]. First, we determined the stability of carbenoxolone in its free-form (Figure 1), to further compare it with the presence of the drug post encapsulation. 

Characterization of carbenoxolone particles showed that Tween 80, which is usually added to stabilize the oil drops that disperse in the water, increased the size of the particles (Figure 2C, Figure S1); In the case of empty particles (no drug) with Tween 80, a diverse size distribution with no valid particle diameter (PDI) was obtained by DLS (Figure S1). SEM images revealed a spherical shape with a ‘hollow-like’ core only in the presence of the drug. Interestingly, this morphological defect was not observed when fabricating the drug-free particles with Tween 80 (polyoxyethylene sorbitan monooleate 80), nor was it detected in our Tween 80-free formulations. A similar phenomenon was reported in PLGA particles fabricated with various surfactants encapsulated with insulin [32], but in that case, the cause was the addition of Span 80 (sorbitan monooleate 80), which is of a low hydrophilic-lipophilic balance (HLB) and is usually added to stabilize the water-in-oil (W/O) emulsion. There the phenomenon was attributed to the migration of the drug aqueous solution droplets towards the outer aqueous phase in the process of emulsification, which was immediately stopped by the hardening of particles [32,33]. 

In our case, it is possible that the drug migrated from the non-stabilized water drops, through the polymer matrix, and to the outer water continuous phase, followed by rapid hardening of the matrix by evaporation. The relatively high loading capacity (~60%) is typical for PLGA microspheres. The cumulative release kinetic profile showed a burst release in the first 5 h, followed by a zero-order release up to day 4, and subsequently by a plateau resulting from complete depletion of the drug (Figure 1C). (For calculations see Appendix A).

To validate the efficacy of the encapsulated drug in-vitro, we performed a bioassay of anoikis, which is increased upon carbenoxolone treatment, as we previously demonstrated in LLC cells [8]. In another study, human thyroid cancer cells that were exposed to a high dose of carbenoxolone (up to 50 µM) were also shown to increase susceptibility to anoikis, which was attributed to the loss of gap junctions [34]. Moreover, patient-derived glioblastoma cells showed increased sensitivity to anoikis when treated with carbenoxolone [35]. In the encapsulated form, carbenoxolone maintains its activity suggesting that at a low dosage, as in our slow-release particles, the drug provides sufficient efficacy (Figure 3). The effect we detected with 100 μg/mL particles (~0.5 μM) of the encapsulated drug was similar to that of 0.1 μM with the free drug as previously shown (22% (Figure 3) and 37% [8] respectively). This could be attributed to the slow release of the drug in the encapsulated form and the lower availability of loaded drug after 72 h in this assay (only ~80% is released after 72 h in complete hydration). Moreover, it is common for encapsulated drugs to require higher dosage in-vitro, compared with the free form, to be more efficient in-vivo as was previously shown [26]. Since cell death in circulation is one of the pivotal stages in the metastatic multi-step process, the ability of the drug to reduce floating cell survival is of great clinical relevance.

To evaluate the efficacy of carbenoxolone particles in preventing lung metastases, we utilized the tail-vein injection model, which is commonly used in studies of metastatic formation [36,37]. This model monitors the ability of cancer cells to circulate and colonize in the lungs, as the cells are injected directly into the circular system. We previously showed that pre-treating mice with carbenoxolone 5 days prior to LLC injections, followed by intraperitoneal (IP) administration of the encapsulated drug, 40 mg/kg every other day over 25 days, resulted in 48% less metastatic formation in the lungs compared with the control group [8]. Moreover, the lesions formed were smaller than those found in the untreated group. These studies showed that the drug has impaired the cells’ adherence to the lung tissue, thereby reducing the formation of metastatic lesions. Here, we recapitulated this experiment, but instead of using free carbenoxolone, we introduced a single systemic administration of slow-release drug-loaded particles of 40 mg/kg carbenoxolone. The drug-loaded particles maintain drug efficacy in-vivo, as demonstrated by thereduction in the number and size of metastatic lung lesions (Figure 4). 

To provide a proof of concept of targeting carbenoxolone treatment to the pulmonary tissue, we delivered the particles also via intranasal administration. A single intranasal treatment of a fixed dose of 1 mg/mouse encapsulated carbenoxolone was administered 5 days prior to IV injections of LLC cells. We found this concentration safe in intranasal administration, as higher concentrations were more viscous and posed a risk for accidental suffocation of the mice. While all treated mice were alive on day 20, two of the controlled mice died at day 19. Gross pathology of these mice revealed detectable metastatic lesions in the lungs, which could explain their death. Images show that 6-coumarin tagged particles were detected clearly in the lungs of the mice as long as 20 days post drug-encapsulated particle administration (Figure 5D). These findings are in agreement with other studies showing the respiratory tract to be successfully used to deliver particles to the lungs [20,21,38]. The efficacy of the slowly released particles was high, as indicated by the lower number and smaller size of metastatic lesions in the lungs of mice compared with both control groups (Figure 5A, Tables S1 and S2). 

In our previous study, the free drug was administered using different LLC in-vivo models; subcutaneous, orthotropic, tail vein and resection, where a repeated administration of the drug was required [8]. Interestingly, we can determine that the encapsulated drug, whether administered IV or intranasally, required a considerably lower dose (<×10) using a single pre-treatment, and resulted with a similar effect to that of repeated dosage of the free drug in our previous study [8].

## 4. Materials and Methods

### 4.1. Stability of Carbenoxolone in Water

The stability of free carbenoxolone in double distilled water was assessed using the calibration of HPLC (System Gold Microbore, Beckman Coulter). Carbenoxolone released fractions were analyzed with Kinetex 5u EVO Column C18, 150 × 3.9 mm. The mobile phase was acetone: PBS 40:60, and the flow rate was 1 mL per minute. The temperature was set to 20 °C and detection was at 254 nm. Elution time was 1.5–1.6 s (Figure 1A). 

### 4.2. Preparation of Drug-Loaded Particles

To fabricate drug encapsulated particles with a mean diameter of ~1 micron, which would be compatible for inhalation, we used two distinct W/O/W formulations of PLGA (50:50, capped, Mw 24,000–38,000), either with or without Tween 80 (HLB = 15) [39] as a stabilizer. Carbenoxolone drug-loaded particles were prepared as previously described [26,40,41]. In short, 100 mg PLGA (Sigma-Aldrich, Cat. No. 739952) with 50:50 lactic-to-glycolic acid ratio were dissolved in 2 mL dichloromethane. A solution of carbenoxolone in PBS (140 mg/mL) was added (200 μL) to the dissolved polymer, and the mixture was homogenized using WiseTis Homogenize (type HG-15D, Witeg, Germany) for 1 min on ice, leading to the formation of the primary emulsion W/O. PVA (Poly-vinyl alcohol) 5% *w/v* of ~67 kDa (Sigma-Aldrich, Cat. No. 81383), saturated in dichloromethane (4 mL), was rapidly added to the emulsion, and the solution was again homogenized. The primary emulsion was then emulsified into 50 mL of 2.5% *w/v* aqueous solution of PVA. The resulting multiple W/O/W was mixed for 5 min, and 2.5 mL of cold isopropanol was added to the W/O/W double emulsion. After 60 min of extensive stirring, the particles were centrifuged and washed three times. After the final wash, the particles were lyophilized (Freezone 6 plus, Labconco, Kansas City, MO, USA) for 48 h, resulting in a fine powder of dry PLGA particles loaded with carbenoxolone. For the preparation of the particles with the surfactant, 4% *v/v* Tween 80 (Thermo Fisher Scientific, Cat. No. BP338-500) was added after the addition of the drug. Empty particles were prepared in the same way, without carbenoxolone. (A more detailed protocol can be found in the Supplementary section). The average yield of particle batch was ~86% *w/w*.

### 4.3. Particle Characterization

The particle samples were imaged and examined SEM (FEI Quanta 200 microscope). A small amount of the sample was spread onto a conductive adhesive carbon tape attached to an SEM grid. A thin film of Pd/Au coating was sputtered onto the sample (SC7620 Sputter coater, UK). The mean diameter of the particles was calculated based on measurements of 30 randomly chosen particles. Size and charge of particles were analyzed using DLS/Zeta sizer nano series (Malvern Instruments, Malvern, UK) at 25 °C in DTS1070 disposable capillary cells. One mL of each sample in 100 µg/mL was injected to measure the size and the zeta potential.

### 4.4. Drug Loading and Release Kinetics

To study the kinetic release of carbenoxolone, we performed dissolution tests as follows: drug-loaded particles (10 mg) were incubated with 1 mL PBS pH = 7.4. The release assay was performed in PBS containing 0.1% solutol-HS15, for particle dispersity. The solution was sampled every 24 h, over the course of 120 h, and analyzed for carbenoxolone concentration using HPLC, after which a cumulative release graph was plotted. Carbenoxolone was detected as a peak at 1.5 min, with 40% acetonitrile in PBS at the mobile phase. The flow rate was 1 mL per minute and the detection monitored at 254 nm wavelength (Figure 1B,C).

### 4.5. Anoikis Assay

Carbenoxolone was previously shown to increase the susceptibility of cells through the induction of apoptosis under non-adherent conditions, i.e., anoikis using poly(2-hydroxyethyl methacrylate) (p-HEMA) coated plates (Sigma-Aldrich, Cat. No. P3932). To evaluate the activity of drug-loaded particles, the same bioassay was applied. Briefly, a solution containing 20 mg/mL p-HEMA in 95% ethanol was prepared and left at room temperature to dissolve for 48 h. Once dissolved, the solution was pipetted into 6-well culture plates. The plates were left half covered in a sterile environment on a rocking plate until the ethanol evaporated and the p-HEMA solidified, coating the plates evenly. The plates were then washed twice with PBS to remove any possible traces of ethanol. Each plate was incubated with growth media containing either empty or carbenoxolone-loaded particles at a concentration of 100 µg/mL. All the plates were seeded with 50,000 LLC cells/well and incubated for 72 h at 37 °C. Cell viability was measured using WST-8 (Sigma-Aldrich, Cat. No. 96992) according to the manufacturer’s protocol, and the cells were incubated at 37 °C for 90 min. Absorbance was measured at 450 nm using a plate reader (Wallac 1420 VICTOR plate-reader, Perkin-Elmer Life Sciences, Waltham, MA, USA) and bright-field images were obtained using an Olympus IX-73 microscope.

### 4.6. Tail Vein In-Vivo Model

Eight-week-old C57BL/6J mice (Harlan, Rehovot, Israel) were pretreated IV with either carbenoxolone loaded particles or control particles of the non-surfactant preparation, 8 mice per group. On the 5th day, all mice have been injected IV with 1 × 10^6^ LLC-GFP cells in 100 μL PBS. The endpoint was set as 21 days after treatment initiation, and all mice were then sacrificed, as in our previously published work [8]. Lung tissue was harvested, weighed, and left in 4% formalin overnight for fixation. Lungs were then transferred to 80% ethanol and a histological serial section was performed. H&E staining of histological sections enabled lesion counting. Images of lungs were taken using a light microscope (Zeiss), and the total number of lesions per slide was counted. 

### 4.7. Intranasal In-Vivo Model

To direct the particles to the lungs of the mice, we used an intranasal delivery of suspended particles. Eight-week-old C57BL/6J mice (Harlan, Israel) were pretreated with carbenoxolone loaded particles, empty particles as a control treatment, or PBS as an additional control. Treatment was administered intranasally by applying 50 µL of a 20 mg/mL solution (equivalent to 25 mg/kg). On the 5th day, all mice have been injected IV with 1 × 10^6^ LLC cells in 100 µL PBS. On day 19, two mice of the two untreated control groups died at which point all the mice were sacrificed. The lungs were harvested, weighed, and fixed in 4% formalin overnight, followed by histological analysis, as detailed in the tail vein experiment (For more details please see Appendix B). 

### 4.8. Lung Biodistribution Study Using Fluorescently Labeled PLGA Particles

To detect pulmonary localization of carbenoxolone PLGA particles following intranasal administration, particles were co-loaded with 6-coumarin, enabling fluorescent imaging. For detection of 6-coumarin particles in the lungs, paraffin-fixed lung sections (as detailed above) were stained with DAPI and imaged using an inverted fluorescent microscope. Non-labeled PLGA particles served as a control to set a fluorescence baseline (Olympus IX73). 

### 4.9. Statistical Analysis

In-vitro data are presented as means ± SD, whereas in-vivo data are presented as means ± SE. Differences in cell viability, the number of lesions, and lung weight were assessed using the unpaired two-tailed Student’s *t*-test, and *p* < 0.05 was considered statistically significant. 

### 4.10. Ethical Approval

All institutional and national guidelines for the care and use of laboratory animals were followed and protocols were approved by the Hebrew University Ein Kerem Medical School IACUC (protocol MD-16-14648-5).

### 4.11. Cell Culture

All cell lines were characterized and purchased from ATCC. Cells were used for experiments up to p20 and were Mycoplasma free, using EZ-PCR Mycoplasma Test Kit (Biological Industries, catalog number 2070020).

## 5. Conclusions

We showed that PLGA carbenoxolone encapsulated particles maintain efficacy in preventing lung metastases when administered in a low dose. Considering our recent demonstration that the combination of carbenoxolone with the anti-angiogenic drug, Bevacizumab, can prolong survival in mice [7], our novel drug delivery system might provide a new platform for a dual administration of combinational therapy that is not restricted to a specific drug. Such localized delivery may improve the safety of drugs that portray grim side effect profiles. 

## Supplementary Materials

The following are available online at https://www.mdpi.com/2072-6694/11/9/1383/s1, Protocol for preparation of particles, Figure S1: DLS analysis of drug-encapsulated and empty particle formulations, Figure S2: Stability of free carbenoxolone vs. encapsulated drug, Table S1: Summary of size of metastatic lesions in the lungs of mice post intranasal administration, Table S2: Summary and *p*-value of size of metastatic lesions in the lungs of mice post intranasal administration, Table S3: Toxicity (weight loss) of mice under treatment with 50 mg/kg of carbenoxolone in its free form.

## Appendix A

### Release Calculations

The loading concentration of carbenoxolone was calculated as 101 μg per 10 mg particles (1% *w/w*). After 48 h, approximately 60% of the original amount of the drug was detected in the solution, using HPLC, and after 96 h only 40% of the original amount of the drug was detected. Release kinetics was calculated based on HPLC determination of the sampled particle aqueous mixture, where carbenoxolone was released from the particles to the aqueous solution (sink conditions). However, notably, disintegration occurred during drug incubation in release medium; this was considered in our calculations.

## Appendix B

### Intranasal Treatment in C57BL/6J Mice

Mice were pre-treated intranasally with 6-coumarin-drug-loaded particles (Empty particles were set as control groups). On the 5th day, all mice were injected intravenously with Lewis lung carcinoma cells (5 × 10^6^). The endpoint was 20 days after treatment initiation due to death of mice of the control groups. The distribution of number and size of lesions in the lungs of mice treated with carbenoxolone loaded particles, empty particles, or control was added to the supplementary section (Tables S1 and S2).
