# Supplementary material for: Encapsulated Carbenoxolone Reduces Lung Metastases
Source: Cancers (Basel). 2019 Sep 17;11(9):1383. doi: 10.3390/cancers11091383 (PMC6771083; doi:10.3390/cancers11091383)
Supplement: Supplementary file 1 [file cancers-11-01383-s001.pdf]

<u>**Supplementary Materials**</u>

Size of the different formulations was analyzed with DLS. Figure S1 summarizes the results.

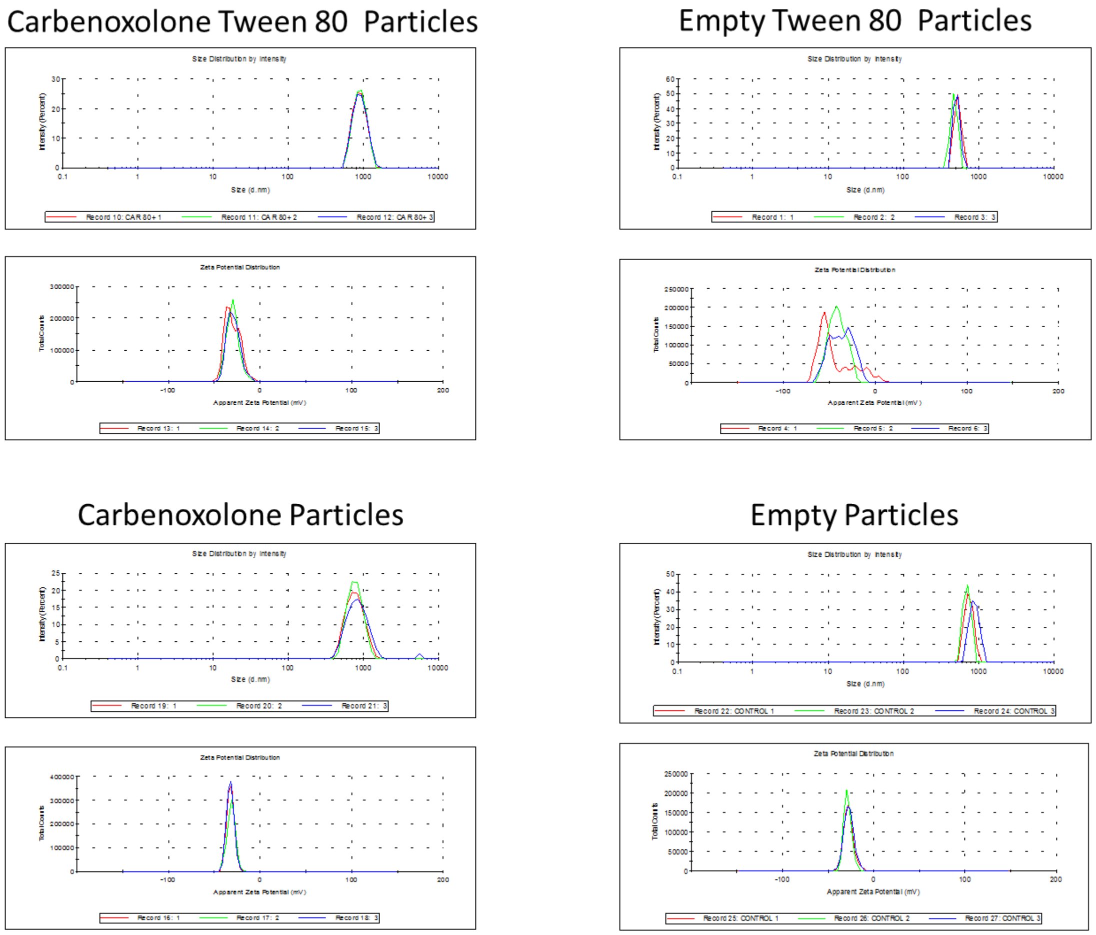

Figure S1: DLS analysis of drug-encapsulated and empty particle formulations.

DLS (Dynamic light scattering) analysis showing Zeta potential and size distributions of all 4 formulations: Empty particles, drug(Carbenoxolone)-loaded particles, Tween 80 empty particles, and Tween 80 drug-loaded particles.

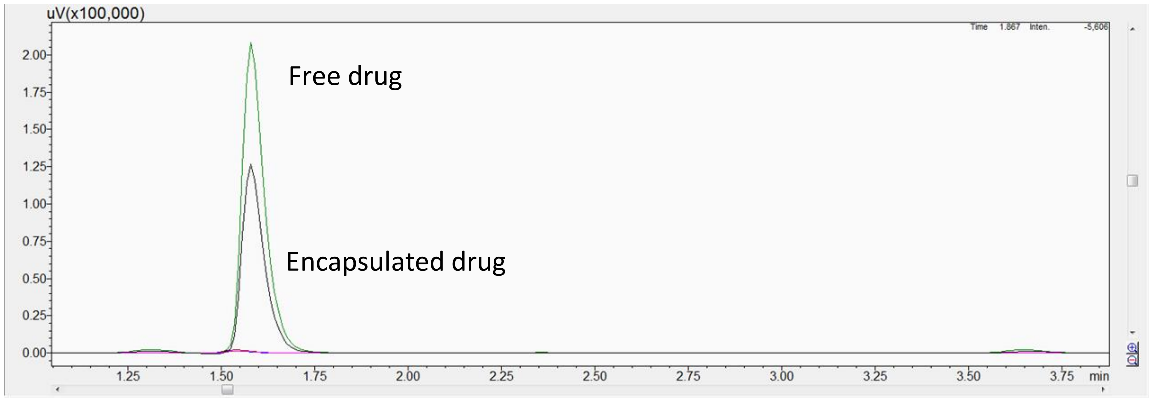

Figure S2: Stability of free Carbenoxolone vs. encapsulated drug: HPLC of Carbenoxolone encapsulated vs. the free form over 96 hours.

Table S1: Summary of Size of metastatic lesions in the lungs of mice post intranasal administration.

Drug loaded particles	<250 µm	250-500 µm	>500 µm	Sum
D11	13	12	20	45
D13	14	11	18	43
D14	21	15	17	53
D15	13	19	24	56
D16	7	10	12	29
D17	16	8	11	35
D18	14	12	20	46
D19	13	6	7	26
Average	13.9	11.6	16.1	41.6

Empty particles	<250 µm	250-500 µm	>500 µm	Sum
E20	24	12	21	57
E21	13	16	32	61
E22	37	19	12	68
E23	25	18	12	55
E24	18	10	25	53
E25	36	12	28	76
E26	17	19	29	65
Average	24.3	15.1	22.7	62.1

Control	<250 µm	250-500 µm	>500 µm	Sum
C1	17	9	24	50
C2	18	17	23	58
C3	28	9	29	66
C4	21	28	35	84
C5	13	8	30	51
C6	16	17	21	54
C7	11	7	38	56
C8	18	11	24	53
C9	23	11	20	54
Average	18.3	13.0	27.1	58.4

Table S2: Summary and p value of Size of metastatic lesions in the lungs of mice post intranasal administration.

Treatment	Average	stdev	SE
Drug loaded particles	41.6	10.8	3.8
Empty particles	62.1	8.1	3.1
Control (naïve)	58.4	10.7	3.6

ttest	
Drug vs. Empty	0.0012
Drug vs. Control	0.0057
Empty vs. Control	0.46

Table S3: Indication Toxicity (weight loss) of mice under treatment with 50 mg/kg of Carbenoxolone in its free form

Treatment	Day 1	Day 2	Day 4	Day 7	Day 8	Weight change [g]	Percentage	Average
Carbenoxolone	28	26	26	23	23	-5	-18%	-6%
Carbenoxolone	29	29	26	28	27	-2	-7%	
Carbenoxolone	30	29	28	29	30	0	0%	
Carbenoxolone	33	32	31	33	31	-2	-6%	
Carbenoxolone	32	29	31	32	33	1	3%	
Carbenoxolone	26	24	25	23	24	-2	-8%	
Cont	32	32	32	32	33	1	3%	3%
Cont	28	28	29	28	29	1	4%	
Cont	31	31	31	31	32	1	3%	
Cont	26	27	28	28	28	2	8%	
Cont	30	30	29	30	32	2	7%	
Cont	29	28	29	29	28	-1	-3%	

Protocol for preparation of drug-loaded and empty particles

PLGA (100 mg, Sigma-Aldrich, Cat. No. 739952) with 50:50 lactic-to-glycolic acid ratio was dissolved in 2 ml dichloromethane. A solution of Carbenoxolone in PBS (140 mg/ml) was added (200 μl) to the dissolved polymer, and the mixture was homogenized using WiseTis Homogenize (type HG-15D, Witeg, Germany) for 1 min on ice, leading to the formation of the first emulsion (W/O). Poly-vinyl alcohol (PVA) 5% w/v of ~67 kDa (Sigma-Aldrich, Cat. No. 81383) saturated in dichloromethane (4 ml) was rapidly added

to the first emulsion, and the solution was homogenized for the second time. The primary emulsion was then emulsified into 50 ml of 2.5% w/v aqueous solution of polyvinyl alcohol (PVA). The resulting multiple W/O/W were mixed for 5 minutes, and 2.5 ml of cold Isopropanol were added to the W/O/W double emulsion. After 60 minutes of extensive stirring, the microspheres were centrifuged and washed three times. After the final wash, the microspheres were lyophilized (Freezone 6 plus, Labconco, U.S.) for 48 hours, resulting in a fine powder of dry PLGA microspheres that contained Carbenoxolone. An additional preparation was done with Tween 80 (Thermo Fisher Scientific, Cat. No. BP338-500), where 84 µl of Tween 80 were added after the addition of the drug. Empty microspheres were prepared in the same way without Carbenoxolone, one batch for each preparation (with and without surfactant). Morphological studies with a scanning electron microscope (SEM) and size distribution Microspheres were conducted for characterization. All particles characterizations and experiments were performed post-lyophilization.
